# RGB-Based Staircase Detection for Quadrupedal Robots: Implementation and Analysis

**DOI:** 10.3390/s25237247

**Published:** 2025-11-27

**Authors:** Piotr Wozniak, Paweł Penar, Damian Bielecki

**Affiliations:** 1 Department of Computer and Control Engineering, Rzeszow University of Technology, 35-959 Rzeszow, Poland; 2 Department of Applied Mechanics and Robotics, Rzeszow University of Technology, 35-959 Rzeszow, Poland; ppenar@prz.edu.pl (P.P.); damianb121@gmail.com (D.B.)

**Keywords:** stair detection, YOLO, deep learning, object detection, quadrupedal robot, mobile robot

## Abstract

In this paper, we present a stair detection algorithm verified on various platforms, including a real quadruped robot. The proposed approach detects dangerous situations, such as proximity to stairs, in real time, enabling the robot to move safely. In our research, we utilized data collected by a moving four-legged robot, recorded in 18 sequences containing more than 21,000 color images from two cameras positioned at different perspectives. To address the challenge, we utilize a deep neural network with RGB inputs for object detection, complemented by preprocessing, and post-processing. A key feature of this approach is its adaptability to varying camera views, including both front and bottom perspectives on the robot, with training that incorporates multi-camera images from both views. We implemented and tested this algorithm on the Unitree Go1 robot, as well as on other embedded platforms. Using the trained YOLO version 11n network on a single sequence and testing on 17 sequences, we achieved an average mAP@50 of 51.30 for images containing only stairs and 87.56 for all images. This method enables early hazard detection during stair navigation. The proposed evaluation scenario tests the model’s adaptation from a single training sequence to multiple unseen sequences, extending existing stair detection methods for quadrupedal robots. The dataset presents high variability in stair appearance due to the robot’s perspective and limited real-time processing capacity.

## 1. Introduction

With rapid advancements in robotics, autonomous robots are being increasingly deployed across a broad spectrum of applications, performing tasks carried out by humans. In-progress intensive research aims to integrate these autonomous systems into dynamic environments further, expanding their capabilities and practical usage. One of the greatest challenges, especially for mobile robots, is ensuring reliable and safe navigation. In complex multi-story buildings, obstacles such as stairs often limit the mobility of wheeled and other ground-based robots. The ability to climb and navigate stairs [1] is essential, as stairs are common in many buildings. Robots must be specifically equipped and engineered to handle these scenarios. Movement on or near stairs is widely discussed in the literature [2,3] and can be a primary objective or part of more complex tasks. Numerous studies have introduced novel mechanisms, sensors, and performance evaluations specifically for stair-climbing robots [3,4]. The type of robot plays an important role in solving the stair climbing problem, and their classifications were widely discussed in [3]. We can define structures that represent a crawler robot [5], a foot robot [6], a wheel-coupler robot [7], and a wheel-legged robot [8]. Effective stair navigation for robots requires early detection of stairs and adaptation of the robot to perform the necessary movements. Sensors play a crucial role in this process, with different approaches employed depending on the type of device. Common methods for stair detection include the use of laser rangefinders [9] and LiDAR [10] sensors for precontact detection, while accelerometers [11] and gyroscopic sensors [12] aid in real-time navigation during ascent or descent. However, research on autonomous mobile robots places significant demands on hardware. Despite the growing adoption of robots, advanced platforms remain a costly component of research. As a result, work can be divided between real environments [13] and virtual environments [14]. A task can be challenging for hardware and often causes failures, so data from virtual environments are used in research.

An important and widely used approach in robotics is computer vision [15]. The use of cameras, such as RGB or RGB-D [16,17], not only complements other sensors, but also facilitates decision-making based solely on visual information. The advantage of such solutions is the number of available camera types, but also mechanisms such as stereoscopic vision [18]. These approaches are still under intensive development, and their main goal is to enable robots to detect stairs at an early stage. The proliferation of deep learning methods, coupled with advances in computational capabilities, has significantly accelerated progress in image analysis. The development of deep learning methods, particularly convolutional neural networks (CNNs) [19], combined with increased computational power, allows precise analysis and interpretation of image content. The early detection of stairs as an element of the environment is in good agreement with the challenges of object detection, as supported by recent studies in the literature [20].

In this paper, we focus on the detection of stairs as an object in the image using a quadruped robot that can mechanically climb stairs. This robot is also a key element in a number of current wide-ranging studies [21]. In the literature, some works utilize this type of robot for navigating various indoor and outdoor environments, addressing the challenge of climbing stairs. Research also addresses exploring the application of quadrupedal robots in scenarios such as disaster response, search and rescue [22], and urban exploration [23], where stairs are common structural elements. Beyond stairs, these robots face various challenges such as navigate and operate autonomously in complex environments [24]. The main challenge appears to be the detection of stairs, addressed through computer vision by leveraging a hardware set-up with cameras positioned at multiple perspectives on the robot’s body. However, most of the datasets on stair detection found in the literature are acquired from perspectives other than those typical for quadruped robots. Consequently, the data sets used for stair recognition must be updated to reflect these different points of view. An important aspect to consider is the use of cameras on the mobile platform that observe the ground directly beneath the robot as it moves. This creates new possibilities for recognition of objects, e.g., stairs, that are inaccessible for classical mobile robots. The most important aspect is that few studies present a real robot that registers and moves directly on the stairs. Verifying such a scenario is crucial for the proper implementation of the proposed solutions in real-world environments.

Existing studies and public datasets do not address the sequential scenario explored in this work, where stairs appear as integral elements of indoor environments observed by a mobile quadruped robot in motion. Unlike static datasets or single-view detection tasks, our setup considers a continuously changing camera pose typical of legged locomotion, where the robot perceives its surroundings in real time and must autonomously identify critical structural features such as stairs while moving through complex indoor spaces. This problem relates to long-term indoor perception in stable environments, where the robot revisits previously seen areas and must rely on persistent landmarks. The collected data, therefore, provide a foundation for developing methods robust to small, imbalanced datasets, serve as a practical extension of more general benchmark-oriented stair detection scenarios, and support future research on loop closure and place recognition, where stairs act as stable reference points within the environment.

In light of the limitations identified in the current existing literature, the principal contributions of this article are as follows:

- evaluation of a staircase detection algorithm using unique multi-camera data with significant variation;
- testing and validation on diverse software and hardware setups;
- real-time implementation on an autonomous
  quadrupedal robot;
- introduction and assessment of a sequential scenario in which the robot perceives and detects stairs while navigating dynamic indoor environments;
- publicly available RGB staircase dataset capturing indoor passages from a quadrupedal robot’s perspective.

This paper addresses staircase detection by mobile robots and reviews related work, focusing on quadrupedal robots and computer vision methods. It describes the mobile platform, the process of collecting dynamic sequences, and the proposed YOLO-based method. Results from multiple sequences are analyzed, highlighting performance metrics and processing times across various platforms and models, and demonstrating the method’s applicability to real-world quadrupedal robotic applications.

## 2. Related Works

The problem of detecting stairs can be considered in the context of implementing effective strategies for gait generation to overcome them by quadruped robots. This, in turn, remains of constant interest for researchers, as indicated, among others, in the review paper [25]. The paper discusses various aspects of quadruped robots, such as types of legs, their construction, gait patterns, and types of movements. The article also presents some considerations for quadruped robots used in climbing applications. In the paper [26], an optimized static gait was proposed. The first element of the algorithm determines the optimized position of a robot standing statically on the stairs. Then, the way in which the robot will move from one position and pose to another position and pose was presented. Additionally, a high-level planning algorithm was proposed to adjust the robot’s step length during the execution of a sequence of movements leading to overcoming stairs. The proposed method was tested only in simulation, unlike the work [27]. There, the proposed algorithm for climbing stairs was tested based on information about their geometry, which was tested on the real Aliengo robot. The presented problem of the gait generation strategy of a quadruped robot is a mechanical domain. However, its effective implementation, understood as maneuvering between gait modes, should be supplemented by solving the problem of ground detection, including stairs. Classical approaches to image-based staircase detection have relied on a variety of techniques, such as line detection. Feature-based methods and gradient-based approaches are used. Early methods for recognizing stairs often used line detection algorithms, such as the Hough transform, to detect edges and contours that correspond to the steps and risers of stairs [28]. Another widely used technique is the Speeded-Up Robust Features (SURF) [29] method, which extracts distinctive features from images, making it robust to changes in scale, rotation, and perspective. Furthermore, histograms of the Oriented Gradient (HOG) [30] have been frequently used for object detection, including the identification of stairs structures, by analyzing gradient information of image regions. Although classical methods were effective for some tasks and relatively computationally undemanding, they often failed in complex real-world environments. In such environments, stairs could appear differently under varying lighting or occlusion conditions.

A highly developed approach to the problem of staircase detection is the use of deep learning. Examples of such tasks related to moving around different terrains are patrolling [31], the general task of jumping varying distances and heights [32], and the adaptive gait pattern adjustment method to overcome unstructured terrains [33]. Recent studies focus primarily on methods and datasets in the field of computer vision and deep learning. Several works in the literature address stair structures, particularly within the context of computer vision [34]. In addition, many datasets incorporate point clouds [35] obtained from depth cameras, allowing a more detailed analysis of stair features and dimensions. This combination of visual and depth data enhances the evaluation of stair structures, providing richer information for applications in robotics and environment perception. In the thematic research on computer vision, different approaches have been introduced and the following categories of data have been identified:

- color images for object detection [36];
- color images with information about detection of stair lines or segments [37];
- depth information as a 3D point cloud [38].

Many works emphasize the use of depth information not only for stair detection [39] but also for determining the position of steps. This also allows for the development of robot interaction with planes in 3D space. However, this approach requires significant computational performance and depth acquisition devices. An important research direction focuses on the use of color images exclusively. The decision on the complexity of the information required for a specific task depends not only on the nature of the problem, but also on the capabilities of the robot’s computing infrastructure. Many robotic platforms designed for various tasks must limit the resources required for navigation and overcoming obstacles such as stairs. Therefore, it is beneficial to use energy-efficient sensors or to select only specific data. It is also important to combine information from several sources [40], which can optimize the relationship between the result and the required performance. Consequently, relying solely on information from color images becomes an appealing option. Color images provide a balance between simple sensor data and complex 3D data. An RGB image, when combined with appropriate deep neural networks, serves as a powerful source of information about the environment. Color images enable the interpretation of environmental elements in a way that closely resembles human perception. This approach is widely applied in various robotic applications, where object detection using computer vision [41] often serves as the foundation for more complex tasks and further research. A popular and efficient method is You Only Look Once (YOLO) [42], which is the main focus of this work. Many works in the literature are the basis for attempting to apply and study YOLO, also for the quadrupedal. In particular, some of them refer to the technique for detecting stairs and robotics [43]. In comparison, traditional handcrafted-descriptor-based detectors, such as those using HOG, SIFT, or SURF features, exhibit clear limitations under varying lighting and viewpoint conditions. In contrast, deep learning-based methods demonstrate superior adaptability, robustness, and scalability, particularly in complex robotic perception tasks. Several previous studies in the literature have also confirmed that handcrafted approaches perform significantly worse than modern deep learning detectors in comparable object detection scenarios [44,45]. This establishes a stronger methodological foundation for the approach adopted in this work and justifies the focus on a deep learning framework based on the YOLO architecture.

While camera-based perception has been widely explored in various domains, its application in embedded robotic systems for dynamic, real-world environments remains relatively limited and challenging, particularly due to the scarce amount of annotated data available from practical scenarios. Therefore, in this work, we focus on developing a robust, real-time vision system capable of reliable object detection under variable conditions. The primary objective is to design and evaluate a YOLO-based framework that addresses these challenges and serves as a foundation for further robotic perception tasks.

## 3. Dataset

**Figure 1:**
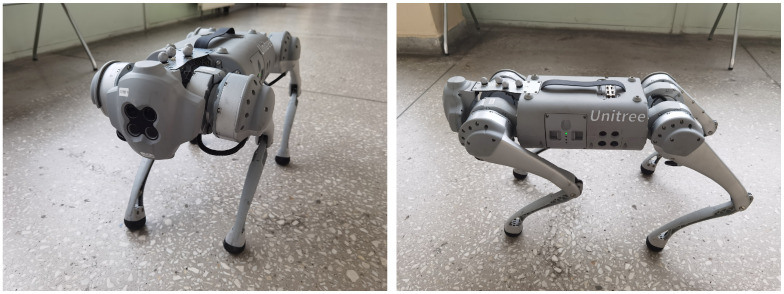
View of the Unitree Go1 robotic platform.

This chapter presents the mobile platform and the data collection process. The paper [3] confirms that quadrupedal robots can overcome various obstacles in the terrain, including stairs. Due to the popularity of such structures, the paper [46] discusses current research issues in key technical areas related to quadrupedal robots. An example of such a platform is the quadruped robot GO1 by Unitree, shown in Figure 1. The manufacturer indicates that the robot has the ability to control the position of each of the 12 joints. This allows for the implementation of force control of the entire robot and the development of speeds up to 3.7 m/s (in the started mode up to 4.7 m/s). Due to the topic discussed, it is important that GO1 has three NVIDIA Jetson Nanos. These are connected by five cameras, which are part of the robot’s sensory system. They are complemented by encoders associated with joints, the IMU system, and distance sensors. In this work, we focus on two cameras that were selected as potentially the most effective in detecting stairs. The front camera is mounted on the robot’s head, whereas the lower front camera is placed under the robot’s head. This choice significantly improved the robot’s ability to observe potential threats both in front of it and below it, especially when the robot approaches stairs. The data collected after preprocessing for benchmarking have been prepared and publicly released (The dataset is publicly available: https://pwozniak.kia.prz.edu.pl/stairsdetection2025, accessed on 20 November 2025).

**Figure 2:**
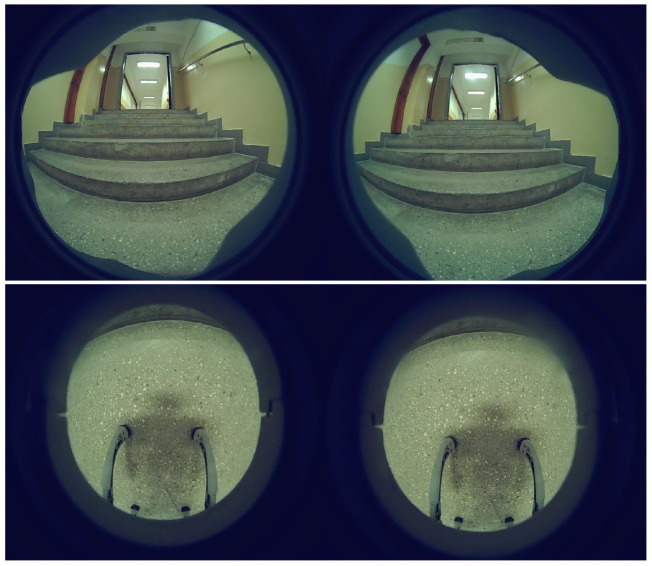
Views from front and front-bottom cameras.

**Figure 3:**
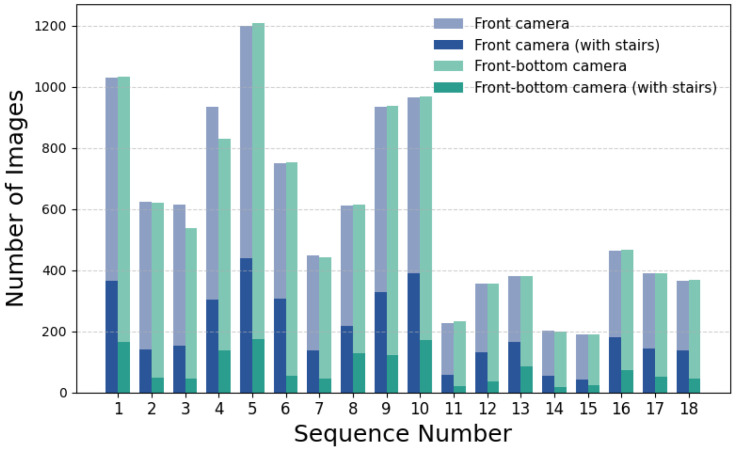
Number of images in sequences for cameras.

The study uses data collected by a Unitree Go1 mobile robot as it navigates through the building environment. The dataset consists of stereo sequences of RGB images captured by cameras mounted on the front and front-bottom of the robot. The raw images captured at 1856 × 800 px are distorted by fisheye lenses, which affect object detection and depth perception. This type of distortion may lead to significant curvature in the image, potentially impacting the interpretation of spatial relationships. An example of the data views from the front and front-bottom cameras is shown in Figure 2. Figure 3 presents a bar chart with the number of images in each sequence and the subset of images containing stairs. The number of images in each sequence varies, primarily based on the length of the route and the data collection unit connected to the robot. An analogous difference occurs in the number of images depending on the camera that records them. The distributed architecture, with cameras operated on different Jetson platforms, causes variations in the number of images captured. The front camera captures a greater number of staircase occurrences due to its position and viewpoint. In contrast, the bottom camera cannot observe the stairs from a distance, but its data are still extremely important. This is because, when stairs are detected, they are typically close to the robot or in direct contact with it. For the front camera, 34% of images contain stairs. For the bottom camera, 13%. Overall, 23% of images from both cameras include stairs.

**Figure 4:**
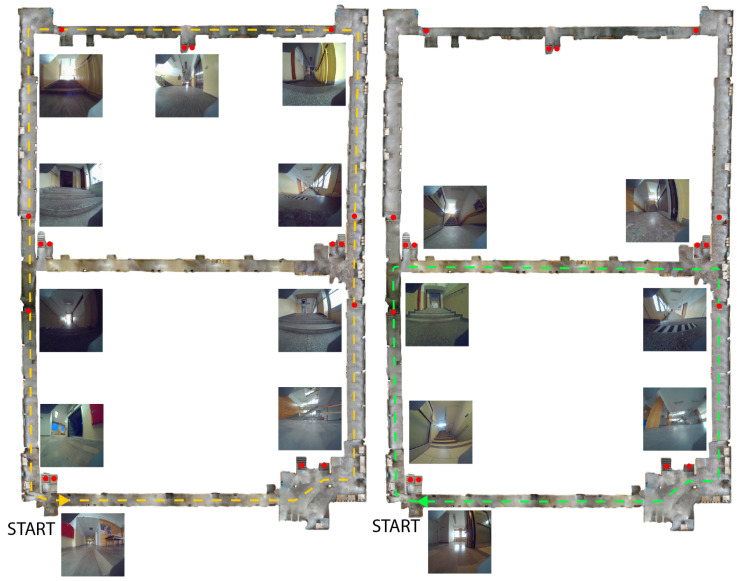
An example of the top view of the robot’s movement path (**left**—sequence 1, **right**—sequence 6).

**Figure 5:**
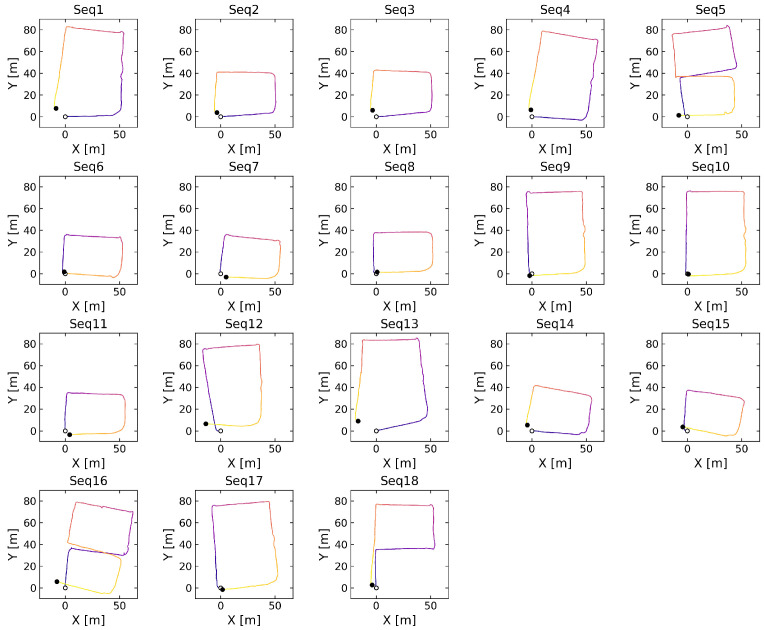
Top view of robot paths using IMU-based localization, with color indicating time progression.

Each image was manually annotated with bounding boxes covering the entire stair surface, unlike some datasets that annotate only the central part. It is crucial not only to detect the stairs but also to assess how much of the scene they occupy from the robot’s perspective. Each labeled image has been scaled to a resolution of 800 × 800 pixels. The robot walked around the building following designated paths. A total of 18 independent sequences were performed, during which the robot climbed the stairs under different conditions. The important conditions that made staircase detection difficult were mainly moving people and changing lighting conditions. The environment in which the robot moved contained sixteen different stairs, and the robot moved directly on six of them. The remaining stairs were also marked and detected, but the robot did not move directly on them. The use of a quadruped robot to obtain the dataset was limited to manual control inside the building. The robot climbed selected stairs using the manufacturer-supplied stair mode, which was manually activated. It is a rather simplified method of movement and is implemented by lifting the limbs to a higher height than in the walking mode. In this way, the robot can climb stairs whose height does not exceed 12 cm. Moreover, such an approach requires that the width of the stairs be appropriate in relation to the robot’s step length. Depending on the direction of the designated route, the same stairs were approached from different points of view. When descending the stairs, the robot was carried to prevent possible damage. The robot also walked on the stairs, avoiding them in some sequences. Figure 4 presents a visualization of the robot’s path from sequences 1 and 6. All stair locations are indicated by red dots, while the robot’s path is represented by a dashed line. In both sequences, the robot initiated its movement from the same starting point. In addition, the figure includes sample images of the stairs captured by the robot at specific locations. The goal of this data collection approach was to enable a learning-from-demonstration mode. In this setup, the mobile robot first performs a supervised traversal of the environment under human guidance and then operates autonomously. Once detached from supervision, the robot independently performs tasks such as object detection and adapts its behavior accordingly, such as when approaching stairs. However, this approach is not without challenges, as the amount of data collected during the supervised traversal is limited. Therefore, it is a challenge to develop solutions in this way. The visualization of the localization based on the IMU data for all 18 sequences is presented in Figure 5.

Compared to publicly available datasets such as StairNet [34] and the RGB-D Stair Dataset [37], the data collected in this study provide a fundamentally different perspective on the stair detection problem. StairNet focuses on egocentric, static images designed for general scene recognition, without robot-mounted viewpoints or sequential data. The RGB-D Stair Dataset, while valuable for multimodal stair detection, contains only static RGB-D pairs recorded under controlled conditions. In contrast, our dataset captures continuous RGB sequences from cameras mounted on a mobile quadruped robot in motion, reflecting realistic variations in viewpoint, motion blur, and fisheye distortion inherent to legged locomotion. Environmental factors such as lighting conditions, stair inclination, and surface texture were intentionally diversified and documented to ensure robustness. Therefore, the proposed dataset complements existing benchmarks by offering a robot-centric, dynamic, and realistic extension of stair detection scenarios relevant to real-world robotic applications.

## 4. Methods

**Figure 6:**
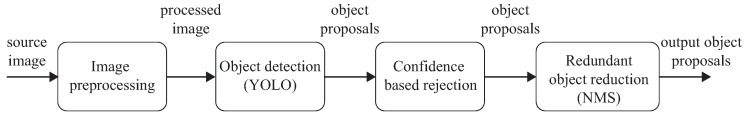
Schematic diagram of the proposed steps for staircase detection algorithm using RGB image.

The proposed method focuses on four main steps. The first was to extract a fragment from a single stereoscopic camera image, followed by post-processing. The second step was object detection using YOLO. This method used the capabilities of the NVIDIA Jetson platform [47] embedded within the robot, enhancing image processing performance and enabling real-time detection. This approach enables simultaneous stair detection and efficient navigation, allowing the robot to perform other tasks in its environment. The third step is to reject low-confidence proposals. This is determined by a global threshold chosen during detector training. The fourth step of the proposed method involves reducing redundant overlapping detected objects. The goal is to eliminate redundant results that might otherwise compromise the accuracy of the operation at this stage. The stages discussed and the result of their activities are presented in the general diagram of Figure 6.

### 4.1. Image Preprocessing

**Figure 7:**
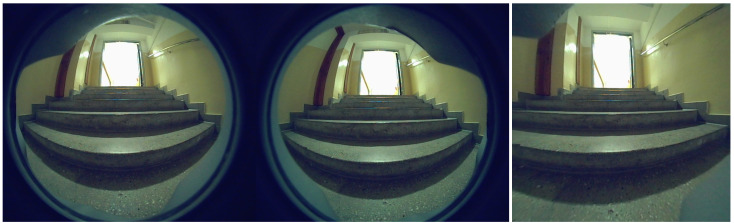
Comparison of source image (**left**) and processed image (**right**).

The processing step involved taking only the left part of the input image for further decision. The fisheye effect was inverted. This operation was based on a field of view (FOV) of 180 degrees and a processed field of view (PFOV) of 120 degrees. The distortion correction parameters for both the FOV and PFOV were manually selected and applied identically to both cameras. The operation was performed to reduce the amount of space covered by the robot body. The effect of processing the raw image is evident in Figure 7. However, after the process was performed, part of the robot’s body was still visible in the image. This was one of the difficulties, especially in the case of the front bottom camera. The final rescaled image has a resolution of 608 × 608 pixels and is fed into the detector input. The decision to preprocess the data was made to reduce the amount of input sent to the network and to minimize distortions that could affect the detection of stairs.

### 4.2. Object Detection—You Only Look Once

**Figure 8:**
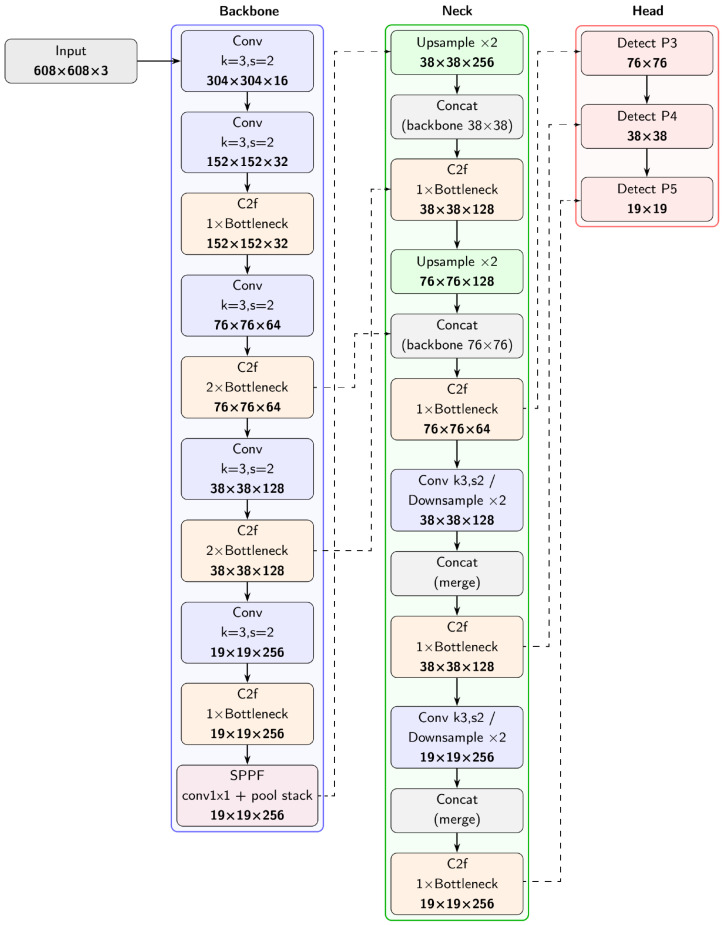
YOLOv11n architecture general scheme used in the experiments.

Based on recent comparative studies [48,49], YOLOv11 has been identified as one of the most accurate and efficient object detection architectures available at the time of this research. Its strong balance between accuracy, speed, and robustness under varying environmental conditions made it particularly suitable for the real-time stair detection problem considered in this study. For comparative purposes, the YOLOv8 variant was also included to assess the performance differences between the latest generation models. The variants of YOLOv8 [50] and YOLOv11 [51] were trained for the detection of stairs based on the images collected. The available pretrained YOLO did not include the detection of stairs, which required training. It was run on an external computing unit supported by a powerful GPU. The input to each network was a 608 × 608 color image obtained by preprocessing. Each network was trained for 50 epochs using the AdamW optimizer, which was selected, with a learning rate of 0.002 and a momentum of 0.9. Data from sequence 1 were randomly divided into training (90%) and validation (10%) subsets. The original network input image resolution was reduced to the chosen input size based on expert assessment of the preprocessed data, ensuring that the resolution was neither too low to lose relevant details nor too high to cause unnecessary computational overhead. During training, mosaic augmentation, horizontal flip, and erasing were used. Before training, the vast majority of the weights, in amounts depending on the network version, were loaded from the model trained on the COCO dataset [52]. The general structure of YOLOv11n is illustrated in Figure 8. The Backbone extracts hierarchical features from the input image through convolutional layers and C2f blocks, with the SPPF block capturing multi-scale context. The Neck fuses features from different backbone levels using upsampling, concatenation, and additional C2f blocks to enhance multi-scale feature representation. Finally, the Head performs multi-scale detection at three different resolutions, predicting bounding boxes, objectness scores, and class probabilities, which enables the network to accurately detect objects such as stairs at various distances and sizes.

The trained models were deployed on the Unitree Go1 robotic platform with NVIDIA Jetson support and on other selected devices. Table 1 presents the properties of the models tested, including the name of the model, the resolution of the input image, the number of layers, the parameters, and the size of the model. Table 2 presents the preprocessing and detection times for different devices and models, measured on four platforms ranging from the Jetson platform running directly on the Unitree Go1 robot, through alternative devices. It also specifies the processor, RAM, GPU model, and Python environment version used during the experiments. Based on the tests, the Jetson platform achieved the shortest detection time with the YOLO11n network. For the ASUS NUC platform and the mobile laptop, the detection times for both YOLO8n and YOLO11n were comparable. Of all the configurations tested, only the mobile laptop consistently achieved a performance in which the preprocessing and detection time was less than or equal to 0.101 s. The ASUS NUC had slightly higher processing times, whereas the Jetson Orin Nano outperformed the Jetson Nano. Three of the four platforms meet the soft real-time threshold of 0.5 s, but the Jetson Orin Nano, despite exceeding this limit, can still be useful when robot speed depends on processing time. In the context of navigation, we consider soft real-time as a processing time that is adequate for control tasks, taking into account the robot’s movement strategy and kinematics, which makes it a practical criterion for evaluating timing in robotic applications.

**Figure 9:**
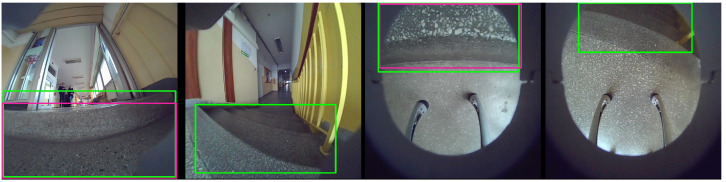
Examples of stair detection (YOLOv11n): green bounding box represents label, and purple bounding box represents detection result.

The differences in model size and detection time are due to the YOLO version used. The YOLOv8n and YOLOv11n versions are faster, with YOLOv11n achieving the shortest detection time on the embedded NVIDIA Jetson platform as well as the smallest model size. This made it an important choice for further studies and direct application on the robot. The example results of the YOLOv11n network trained in Sequence 1 (front camera) are shown in Figure 9.

The first two images show the results of stair detection from the front camera, while the following two images show the results from the front-bottom camera. The first and third results demonstrate successful detections, where the method correctly identified the stairs. In contrast, the second and fourth cases are false detections, where the model did not identify the presence of stairs. This highlights significant errors that can occur even in the vicinity of stairs.

### 4.3. Remove Duplicates Detection

**Figure 10:**
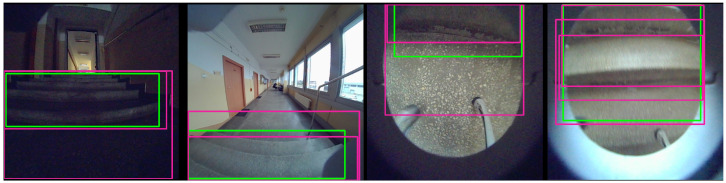
Stair detection examples (YOLOv11n) from front and front-bottom cameras with duplicates.

To minimize redundancy in the staircase detection results, duplicate predictions were removed. This overlap required consolidation to improve accuracy. This was achieved using nonmaximum suppression (NMS) [42] with an intersection over union (IoU) threshold of 0.5, which means that bounding boxes with 50% or more overlap were treated as duplicates and filtered out. The NMS threshold value was selected based on an analysis of the validation subset from Sequence 1 to achieve an optimal balance between detection precision and recall. Figure 10 illustrates sample images with detection results from YOLOv11n, trained on data from the front and front-bottom cameras of Sequence 1. These images demonstrate situations where there are an excessive number of detections for a single object.

## 5. Results

The detailed results of stair detection in Table 3 present the mean average precision (mAP) at thresholds of 0.5, 0.75, and 0.95, along with the corresponding F1 scores, all calculated based on IoU thresholds. In this study, robot walk-through data from sequence 1 was used for training. Images from the first sequence were also used to determine the optimal detection confidence threshold, which was set using an iterative method at 0.25. The results are reported for all sequences, without the training sequence. This approach assumes that the robot initially moves under human supervision, after which it navigates autonomously within the environment under varying conditions, such as lighting and altered views of the stairs. The highest mAP scores were obtained by the YOLOv8n network, with average scores of 82.56, 80.34, and 75.90 (with IoU thresholds of 0.5, 0.75, and 0.95, respectively) for all test sequences. The YOLOv11n network showed similar performance, with mAP scores of 82.21, 79.88, and 75.38. Due to its comparable detection accuracy and shorter image processing time for NVIDIA Jetson, YOLOv11n was selected for further testing on the robot. The observed similarity in mAP values between YOLOv8n and YOLOv11n indicates that both architectures are comparably effective in feature extraction for the stair detection task. However, YOLOv11n provides more efficient inference on embedded hardware, offering lower latency and reduced computational cost, which makes it a more practical choice for real-time robotic applications.

Table 4 shows the average results for all test sequences from 2 to 18. Different scenarios were tested to evaluate the impact of training with data from different cameras. One variant is, e.g., training on data from the front camera only and testing with data from the front-bottom camera. A fusion approach that combined data from two cameras was also used to improve the reliability of stairs detection, making it less dependent on camera type and viewing angle. This multi-camera integration represents a novel aspect of the study, aiming to leverage complementary visual information to reduce the natural imbalance present in the dataset and enhance the model’s generalization capability. The results indicate that training the network on images from both cameras does not necessarily enhance the final outcome. Additionally, using two models is not acceptable due to the computational limitations of the robot. To address this, we implemented NMS for result reduction, which improved the performance of a single model for sequences captured from both the front and bottom front cameras. The best results for the two cameras are highlighted in bold in the table, with most of the highest values achieved by the proposed method. For the front camera, the result was 82.35, and for the front-bottom camera, 92.78 mAP@50. The average result for both cameras was obtained at level 87.56 of mAP@50.

Although the data collected by the robot reflect real scenarios, it is not balanced. The imbalance arises from differences in camera perspectives and the fact that images containing stairs are less frequent than those without them. This imbalance could affect the representativeness of the detection results. To address this, an additional Table 5 was included to present results only for images labeled with stairs. Similarly to the previous table, the incorporation of images from both cameras and the application of NMS yielded the best results. However, when focusing specifically on images labeled with stairs, a noticeable drop in detection performance is observed compared to the results across all images. The average result for the front and front bottom cameras was 51.3 mAP@50.

Comparing YOLOv11n with YOLOv8n, both networks achieved similar mAP scores across all sequences. However, YOLOv11n provided faster inference times on embedded platforms, making it more suitable for real-time deployment on the quadruped robot. This demonstrates that model selection for embedded applications must balance detection accuracy with computational efficiency.

The difference in detection accuracy between the front and bottom cameras mainly results from differences in scene visibility. The bottom camera provides a clearer close-range view of the stairs but is more affected by lighting changes and motion blur, which can slightly reduce detection stability. In contrast, the front camera captures a wider scene but often misses lower stair segments, explaining the observed performance gap. The lower mAP observed when using only one camera, especially the bottom camera, is partially due to the unequal number of stair images between viewpoints and the higher susceptibility of the bottom camera to motion blur and lighting changes. This highlights the importance of multi-camera data fusion and careful threshold tuning for stable detection.

These results highlight the importance of viewpoint diversity, dataset balance, and processing constraints when designing stair detection systems for real-time robotic operation. They also suggest that multi-camera fusion, NMS, and calibrated confidence thresholds are key strategies to improve detection robustness and reduce false negatives, particularly in sequences with limited stair coverage.

**Figure 11:**
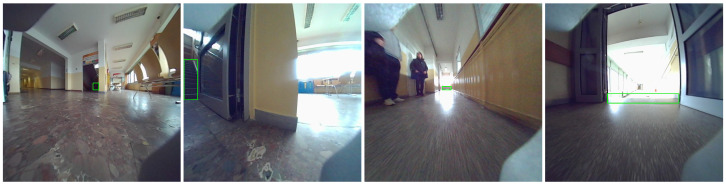
Examples of challenging stair detection scenarios.

However, in this variant, a significant improvement was observed for the bottom-front camera. The initially poorer results may have been due to the limited number of staircase representations captured from this view. Additional training on the complete set of images helped mitigate this issue. The lower result also includes stairs that are far from the robot and do not have direct contact. The relatively low result for the only stairs variant can be attributed to the high difficulty of this subset. Many of the images contain stairs that appear as distant or partially visible objects, often without direct contact with the robot. These cases are more challenging to detect accurately and significantly influence the overall mAP score. To illustrate such problematic examples, Figure 11 shows typical detection difficulties observed in these scenarios.

It is also worth noting that the obtained results are substantially lower compared to average detection performance on standard benchmarks such as the COCO dataset, where state-of-the-art object detectors typically achieve mAP values in the range of 50–60% on the validation set [51]. This discrepancy can be explained by the inherent differences between the two datasets: COCO contains diverse, well-annotated everyday objects captured from multiple viewpoints and distances, while the dataset used in this work focuses on a single object category—stairs—often captured under challenging conditions, including partial visibility, long distances, and limited texture cues. Consequently, the evaluation results on the proposed dataset reflect the specific difficulty of detecting such structures rather than a general decrease in detector performance.

## 6. Discussion

Mobile robots vary in design but often struggle with challenging terrains, especially stairs. Although research on stair detection is crucial, the optimal method for their detection by robots is not yet clearly defined. Image analysis is a crucial but sometimes limited approach due to environmental variability and camera constraints. Color cameras are affordable and common but lack spatial awareness, which depth cameras provide at higher computational and energy costs. Many studies focus on stair detection using geometric properties [4,15]. Consequently, the results of our study are not directly comparable to these types of detection methods. Studies using only RGB cameras for stair recognition align with our approach, showing comparable detection performance [22,53]. However, a precise comparison of these results is difficult because the studies use different datasets for training and testing. Another challenge in comparing our results with those of other researchers lies in the image acquisition methodology. To the best of our knowledge, the dataset employed in this study is unique in the existing literature, as it consists of images captured by cameras integrated into a quadruped robotic platform, offering distinctive perspectives during movement.

The incentive to work on image-based stair detection comes from the Unitree Go1 platform, which uses cameras positioned at different angles to capture the environment and obstacles from multiple perspectives. However, accurate detection alone does not guarantee safe and reliable robot movement. Instead, it should be regarded as one component within a more comprehensive navigation and control system. Furthermore, under difficult environmental conditions, the color image-based detection method may be inaccurate. This limitation underscores the necessity of integrating additional sensors or employing sequential image analysis to enhance detection capabilities. Such improvements would facilitate more robust navigation and maneuvering, ensuring that the robot can effectively navigate complex terrains. All of these issues pose a major challenge in further research.

Moreover, proposed models for stair detection often exhibit limited generalization capability, especially when training is conducted using data from a single sequence. This constraint limits the direct application of such models to other stair detection datasets. Future research in this field should therefore consider multi-domain fine-tuning based on datasets collected from multiple sequences, as well as the integration of additional cameras with varying specifications. These enhancements are expected to improve the overall reliability and adaptability of stair detection models across diverse environments and sensor configurations.

In terms of soft real-time performance, stair detection as a single-task process has moderate timing requirements. While execution times on some platforms exceed typical real-time thresholds, they remain adequate for practical use. However, relying solely on object detection without detailed information (e.g., step positions) is inherently limited and can be demanding for platforms with constrained computational resources. Further optimization may be needed for more complex or multi-task robotic scenarios.

## 7. Conclusions

This paper presents a new staircase detection algorithm for a quadruped robot, designed to improve safety and autonomy in an indoor environment. The approach uses images from two cameras with different perspectives and a deep neural network with RGB input. The method is complemented with non-maximum suppression to reduce duplicate detections. Implemented on the Unitree Go1 platform, the algorithm achieved a mAP@50 of 51.30 for images labeled only with stairs and 87.56 for all images. The results of this work indicate that the use of image data from multiple cameras improves the accuracy of stair detection, allowing earlier detection of this structure. Relying on images from different perspectives may be an option in the case of limited access to larger amounts of data. Compared to other datasets used for object detection, the dataset employed in this study is relatively small. However, the method of its acquisition reflects a realistic operational scenario of a mobile robot focused on stair recognition. From this perspective, the dataset can be considered sufficient for the intended application. A limitation, nevertheless, lies in its relatively low level of diversity, which may reduce its generalization capability when applied to stair detection from different viewpoints or under outdoor conditions. The findings of this work are encouraging for further research on robot control in a stair environment. They can also serve as a basis for working on collision-free robot movement on stairs. In addition, the algorithm was tested on various embedded and mobile computing platforms. The processing time for three of them indicates their suitability for soft real-time implementations, with a processing time limit of 0.5 s. Such a processing time for the robot’s environment information appears sufficient for selecting an effective movement strategy. It is also appropriate in the context of the robot’s kinematic properties and thus useful for control. This demonstrates that, despite its limitations, the proposed solution is suitable for accomplishing navigation tasks for quadruped robots.

## Figures and Tables

**Table 1:** Properties of models used for detection.

Model	Input	Layers	Parameters	Model
	(px)		(M)	(MB)
YOLOv8n	608 × 608	249	2.6	10.4
YOLOv8s	608 × 608	249	9.8	37.6
YOLOv11n	608 × 608	319	2.5	10.0
YOLOv11s	608 × 608	319	9.4	36.1

**Table 2:** Preprocessing and detection times for different devices and models.

			AVG Time (s)			
Device	Specification	Model	Preprocessing	Detection	Total	FPS
NVIDIA Jetson Nano	ARM Cortex-A57, RAM 4 GB, Python 3.8	YOLOv8n	0.224 ± 0.007	0.998 ± 0.141	1.222	0.81
		YOLOv8s	0.225 ± 0.013	2.169 ± 0.134	2.394	0.39
		YOLOv11n	0.230 ± 0.013	0.945 ± 0.161	1.175	0.87
		YOLOv11s	0.222 ± 0.008	2.144 ± 0.161	2.366	0.40
NVIDIA Jetson Orin Nano	ARM Cortex-A78AE, RAM 8 GB, NVIDIA Ampere GPU, Python 3.10	YOLOv8n	0.105 ± 0.001	0.119 ± 0.005	0.224	4.59
		YOLOv8s	0.102 ± 0.001	0.283 ± 0.011	0.385	2.64
		YOLOv11n	0.106 ± 0.001	0.118 ± 0.008	0.224	4.63
		YOLOv11s	0.095 ± 0.007	0.274 ± 0.012	0.369	2.91
ASUS NUC	Intel Core i3-1315U, RAM 16 GB, Python 3.10	YOLOv8n	0.045 ± 0.001	0.069 ± 0.004	0.114	9.43
		YOLOv8s	0.043 ± 0.000	0.180 ± 0.005	0.223	4.52
		YOLOv11n	0.044 ± 0.005	0.071 ± 0.008	0.115	9.26
		YOLOv11s	0.041 ± 0.001	0.176 ± 0.009	0.217	4.72
Mobile Laptop	AMD Ryzen 5 6600H, RAM 16 GB, NVIDIA GeForce RTX 3060, Python 3.12	YOLOv8n	0.033 ± 0.004	0.030 ± 0.008	0.063	16.95
		YOLOv8s	0.031 ± 0.002	0.070 ± 0.009	0.101	10.42
		YOLOv11n	0.032 ± 0.002	0.032 ± 0.004	0.064	15.63
		YOLOv11s	0.031 ± 0.003	0.069 ± 0.009	0.100	10.49

**Table 3:** Detection results using YOLO networks trained on Seq1 for all images (front camera).

Model	Metric	Seq2	Seq3	Seq4	Seq5	Seq6	Seq7	Seq8	Seq9	Seq10	Seq11	Seq12	Seq13	Seq14	Seq15	Seq16	Seq17	Seq18	Mean
YOLOv8n	mAP@50	**94.29**	94.37	**91.66**	83.31	66.53	79.53	84.40	82.44	**83.95**	82.38	**75.56**	**79.47**	85.07	87.43	**75.05**	**79.67**	78.49	**82.56**
	mAP@75	**91.40**	91.19	88.24	79.92	65.87	78.64	76.47	80.30	**80.75**	81.94	**74.58**	**75.92**	84.08	**86.91**	**74.09**	**78.64**	76.85	**80.34**
	mAP@95	**87.38**	**86.95**	**81.34**	74.25	63.13	75.17	74.75	74.89	**73.14**	79.52	**70.37**	**70.26**	79.85	83.77	**71.29**	**72.12**	72.19	**75.90**
	F1@50	94.29	94.48	90.75	86.87	68.70	82.77	**86.33**	84.25	**85.20**	80.84	76.41	**78.60**	85.66	88.15	**74.74**	**81.67**	78.89	**83.45**
	F1@75	88.00	87.93	83.40	**77.35**	65.64	**78.08**	68.95	78.03	76.98	79.00	73.27	71.49	83.25	**85.78**	71.90	**78.77**	73.79	**77.74**
	F1@95	79.90	**79.45**	69.09	64.54	58.98	70.25	64.71	65.85	**60.80**	74.01	64.33	**60.00**	**74.63**	78.97	65.81	**64.62**	63.56	**68.21**
YOLOv8s	mAP@50	93.97	93.88	91.28	82.85	**70.47**	80.87	**85.05**	82.82	79.76	**83.70**	70.93	78.03	81.09	86.39	74.62	78.39	79.59	81.98
	mAP@75	90.51	90.86	**88.66**	79.34	**68.27**	78.52	**77.94**	80.88	76.97	**82.60**	70.51	75.79	80.85	85.60	73.55	77.49	77.53	79.76
	mAP@95	**87.38**	85.48	80.75	74.17	**65.07**	75.39	**75.33**	75.16	70.96	**79.96**	68.40	69.47	77.86	82.46	71.08	71.23	72.47	75.45
	F1@50	94.66	93.88	**91.43**	86.34	71.62	81.62	85.65	82.95	81.69	**82.09**	72.61	77.59	81.09	87.78	74.19	78.35	**81.00**	82.62
	F1@75	87.45	87.66	**85.06**	75.93	**66.44**	75.91	70.75	78.56	74.98	**79.88**	70.65	72.46	80.60	85.51	71.61	76.09	**74.98**	77.32
	F1@95	**80.55**	76.84	68.98	64.08	**59.07**	69.57	64.87	**65.92**	60.77	**74.01**	**65.17**	59.30	**74.63**	78.53	**66.24**	63.17	**63.84**	67.97
YOLOv11n	mAP@50	93.73	**94.45**	91.23	85.27	67.33	**82.33**	72.79	**85.33**	83.95	80.62	75.42	75.92	**88.81**	**88.22**	73.01	78.64	**80.55**	82.21
	mAP@75	90.92	**91.27**	88.45	80.34	65.13	**80.20**	69.93	**82.87**	80.64	79.96	73.74	73.55	**87.56**	86.13	71.51	77.37	**78.36**	79.88
	mAP@95	87.22	86.62	80.91	**75.04**	63.53	**76.62**	67.57	**76.18**	72.72	77.75	70.22	67.24	**81.59**	**84.29**	69.25	71.74	**73.01**	75.38
	F1@50	93.76	**94.93**	90.78	**88.78**	66.98	**82.70**	84.97	**85.40**	85.14	80.03	**76.50**	75.88	**88.56**	**89.35**	74.59	79.03	79.59	83.34
	F1@75	87.83	**87.98**	84.88	75.67	61.91	77.96	72.44	**79.60**	**77.33**	77.97	72.28	70.61	**86.07**	84.47	70.32	76.13	73.79	77.48
	F1@95	80.23	78.63	**69.30**	63.86	58.53	**70.25**	62.64	65.81	60.56	73.13	64.79	57.54	74.13	**80.10**	64.95	64.45	62.83	67.75
YOLOv11s	mAP@50	93.65	92.41	90.37	**85.52**	70.07	75.28	76.06	81.75	75.62	76.65	70.79	77.50	80.85	85.08	72.69	76.47	74.11	79.70
	mAP@75	90.92	89.89	87.17	**81.01**	68.00	74.05	75.49	79.46	72.15	75.55	70.08	75.13	80.85	83.77	71.29	75.45	72.33	77.80
	mAP@95	87.22	84.26	79.95	74.79	64.73	71.36	72.22	74.46	67.96	74.89	67.28	67.76	77.11	80.63	67.74	70.33	68.63	73.61
	F1@50	**94.42**	93.31	90.03	85.63	**72.81**	77.22	80.99	82.52	79.79	77.17	70.51	77.94	80.93	86.04	73.01	77.07	74.96	80.84
	F1@75	**88.26**	87.44	83.12	75.72	65.51	73.08	**77.40**	76.96	70.24	74.45	69.10	**72.89**	80.93	82.90	69.46	74.51	70.59	76.03
	F1@95	80.39	76.02	68.13	62.94	57.33	67.11	**68.41**	65.85	58.90	70.93	63.48	58.07	73.13	76.27	62.15	63.68	62.19	66.76

**Table 4:** Results from various combinations of stairs detection tests across all images (f—front, fb—front-bottom).

			AVG (%)					
Model	Train Seq	Test Seq	mAP@50	mAP@75	mAP@95	F1@50	F1@75	F1@95
YOLOv11n	Seq1 (f)	Seq2-18 (f)	82.21	79.88	75.38	**83.35**	**77.48**	67.75
	Seq1 (f)	Seq2-18 (fb)	87.73	87.37	86.91	87.36	86.62	85.67
	Seq1 (fb)	Seq2-18 (f)	60.94	60.26	60.11	62.47	57.91	56.92
	Seq1 (fb)	Seq2-18 (f)	89.63	89.32	88.32	90.40	89.11	**86.75**
	Seq1 (f & fb)	Seq2-18 (f)	80.94	78.89	74.73	82.35	76.92	67.78
	Seq1 (f & fb)	Seq2-18 (fb)	91.24	90.65	89.04	**93.12**	90.65	86.29
YOLOv11n + NMS	Seq1 (f & fb)	Seq2-18 (f)	**82.35**	**80.00**	**75.63**	82.12	77.02	**67.84**
	Seq1 (f & fb)	Seq2-18 (fb)	**92.78**	**91.91**	**89.89**	92.94	**90.83**	86.30

**Table 5:** Results for various combinations of stairs detection tests on images labeled with stairs (f—front, fb—front-bottom).

			AVG (%)					
Model	Train Seq	Test Seq	mAP@50	mAP@75	mAP@95	F1@50	F1@75	F1@95
YOLOv11n	Seq1 (f)	Seq2-18 (f)	50.26	43.06	29.47	**53.52**	**35.66**	6.29
	Seq1 (f)	Seq2-18 (fb)	16.31	13.19	9.89	13.45	7.03	0.23
	Seq1 (fb)	Seq2-18 (f)	11.61	9.65	9.27	16.33	2.87	0.01
	Seq1 (fb)	Seq2-18 (fb)	24.12	21.61	13.44	30.03	20.05	0.67
	Seq1 (f & fb)	Seq2-18 (f)	46.65	40.29	27.88	50.61	34.21	6.95
	Seq1 (f & fb)	Seq2-18 (fb)	39.38	34.80	22.57	**53.93**	34.27	1.09
YOLOv11n + NMS	Seq1 (f & fb)	Seq2-18 (f)	**50.60**	**43.43**	**30.40**	49.97	34.51	**7.12**
	Seq1 (f & fb)	Seq2-18 (fb)	**52.00**	**44.90**	**29.48**	52.64	**35.63**	**1.11**

## Data Availability

The dataset used in this study is publicly available. It was prepared after preprocessing for benchmarking and can be accessed at: https://pwozniak.kia.prz.edu.pl/stairsdetection2025 (accessed on 20 November 2025).
